# Prognostic factors for survival after bronchoscopic intervention in patients with airway obstruction due to primary pulmonary malignancy

**DOI:** 10.1186/s12890-020-1095-0

**Published:** 2020-02-27

**Authors:** Bo-Guen Kim, Beomsu Shin, Boksoon Chang, Hojoong Kim, Byeong-Ho Jeong

**Affiliations:** 1 Division of Pulmonary and Critical Care Medicine, Department of Medicine, Samsung Medical Center, Sungkyunkwan University School of Medicine, 81 Irwon-ro, Gangnam-gu, Seoul, 06351 South Korea; 2 Department of Internal Medicine, Yonsei University Wonju College of Medicine, Wonju, South Korea; 3 Department of Pulmonary and Critical Care Medicine, Kyung Hee University Hospital at Gangdong, School of Medicine, Kyung Hee University, Seoul, South Korea

**Keywords:** Airway obstruction, Lung cancer, Bronchoscopic intervention

## Abstract

**Background:**

Malignant central airway obstruction (MCAO) occurs in 20–30% of patients with primary pulmonary malignancy. Although bronchoscopic intervention is widely performed to treat MCAO, little data exist on the prognosis of interventional bronchoscopy. Therefore, we evaluated the clinical outcomes and prognostic factors of bronchoscopic interventions in patients with MCAO due to primary pulmonary malignancy.

**Methods:**

This retrospective study was conducted at a university hospital and included 224 patients who received interventional bronchoscopy from 2004 to 2017, excluding patients with salivary gland-type tumor. A multivariable Cox proportional hazard regression analysis was used to identify independent prognostic factors associated with survival after the first bronchoscopic intervention.

**Results:**

Among 224 patients, 191 (85.3%) were males, and the median age was 63 years. The most common histological type of malignancy was squamous cell carcinoma (71.0%). Technical success was achieved in 93.7% of patients. Acute complications and procedure-related death occurred in 15.6 and 1.3% of patients, respectively. The median survival time was 7.0 months, and survival rates at one year and two years were 39.7 and 28.3%, respectively. Poor survival was associated with underlying chronic pulmonary disease, poor performance status, extended lesion, extrinsic or mixed lesion, and MCAO due to disease progression and not receiving adjuvant treatment after bronchoscopic intervention.

**Conclusions:**

Interventional bronchoscopy could be a safe and effective procedure for patients who have MCAO due to primary pulmonary malignancy. In addition, we found several prognostic factors for poor survival after intervention, which will help clinicians determine the best candidates for bronchoscopic intervention.

## Background

The development of new anti-cancer drugs and imaging techniques, such as low-dose computed tomography (CT), has improved the survival rate in patients with primary pulmonary malignancy [1–3]. However, lung cancer is still one of the most fatal malignancies, and many patients die of complications related to disease progression [4, 5]. In particular, central airway obstruction is one of the major complications and causes hemoptysis, atelectasis, and obstructive pneumonia. Eventually, these patients experience disability in daily activities and are at increased risk of repeated hospitalization for respiratory failure [6].

Malignant central airway obstruction (MCAO), which is defined as ≥50% occlusion of the cross-sectional area of the central airway, occurs in 20–30% of patients with primary pulmonary malignancy caused by endoluminal metastasis or extrinsic compression of bulky lymphadenopathy [7]. In patients with MCAO, surgical interventions are rarely indicated for tumors obstructing the trachea and main carina [8]. The effects of chemotherapy or radiation therapy may take a long time to relieve the MCAO [9]. However, bronchoscopic interventions, such as mechanical debulking, laser cauterization, and stent placement, can immediately relieve symptoms related to airway obstruction and improve quality of life [10]. Previous studies have demonstrated the efficacy of this procedure in decreasing the frequency and severity of dyspnea and improving other clinically relevant endpoints [8, 11–13]. In multicenter studies, more than 90% success rates and low complication rates have been confirmed following interventional bronchoscopy in patients with MCAO [14]. However, to the best of our knowledge, few articles have reported the prognostic factors associated with survival after bronchoscopic intervention.

Therefore, the purpose of our study was to evaluate the clinical outcomes and prognostic factors of bronchoscopic interventions in patients with MCAO due to primary pulmonary malignancy.

## Methods

### Patients

The characteristics of airway obstruction were assessed by CT and, when possible, flexible bronchoscopy. MCAO was defined as ≥50% occlusion of the cross-sectional area of the central airway (from trachea to lobar bronchus) on CT or bronchoscopic findings [14]. From January 2004 to December 2017, interventional bronchoscopy was performed in 295 patients with MCAO due to primary pulmonary malignancy at Samsung Medical Center, a university-affiliated hospital in South Korea. Patients with salivary gland-type tumors (*n* = 71), such as adenoid cystic carcinoma, mucoepidermoid carcinoma, and epithelial-myoepithelial carcinoma, were excluded from this study because of their biological differences and good prognosis due to being distinguished from non-small cell and small cell lung cancer [15–18]. Finally, total 224 patients were analyzed.

The Institutional Review Board of Samsung Medical Center approved this study (2019–03-002) and waived informed consent due to its retrospective nature.

### Airway interventional techniques

Interventional bronchoscopy was performed according to standard techniques [19, 20]. Details of airway interventional techniques were described in previous reports [21]. In short, after induction of general anesthesia and intubation with a rigid bronchoscope tube (Bryan Co., Woburn, MA, USA or Karl-Storz, Tuttlingen, Germany), various combinations of airway intervention techniques (mechanical debulking, laser, and insertion of silicone stents) were used depending on the characteristics of airway obstruction. Any intraluminal mass was removed mechanically using rigid bronchoscope tubes and cauterized by using neodymium-doped yttrium aluminum garnet laser (LaserSonics, Milpitas, CA, USA) or diode laser (Biolitec, Ceralas®, Germany). Additionally, if there was an extrinsic compression or high likelihood of rapid tumor ingrowth, a silicone stent (Natural stent [M1S Co., Seoul, Korea] or Dumon stent [Novatech, La Ciotat, France]) was inserted to maintain airway patency [21].

Technical success was assessed based on the anatomic criteria of reopening of the airway lumen to > 50% of the normal cross-sectional area and connection to a viable area in the distal lung on bronchoscopy [14]. If a physician successfully reopened a proximal airway but discovered distal lesions occluding all segmental or subsegmental levels, we classified this as a technical failure [14].

### Data collection

We retrospectively analyzed the medical data from 224 patients using the following data: demographic characteristics, histological type of malignancy, bronchoscopic findings (site of lesion, type of obstruction, and severity and length of stenosis), pre- and post-procedure treatment modalities, airway intervention techniques, procedure-related complications, and survival period.

The following terms were defined identically to our previous report [21]. Poor performance was defined as ≥ American Society of Anesthesiologists (ASA) physical status class 3, which means severe systemic disease with functional limitations [22]. We evaluated the severity of airway stenosis using the Myer-Cotton stenosis grading system: Grade I, ≤50% luminal stenosis; Grade II, 51–70% luminal stenosis; Grade III, 71–99% luminal stenosis; Grade IV, no lumen [23]. Respiratory distress was defined as decrease in oxygen saturation or worsened dyspnea after the procedure and need for additional oxygen for at least 24 h [21]. We defined excessive bleeding as procedure-related bleeding in which blood transfusion or escalated care was needed [21]. Because most patients with MCAO due to primary pulmonary malignancy were already far advanced at the time of the first bronchoscopic intervention, the status of malignancy was defined as follows [21]. The first category was when the MCAO was discovered along with the initial diagnosis of primary pulmonary malignancy that had never been treated, and the second one was when the MCAO was caused by progression of a primary pulmonary malignancy that had already been treated with anti-cancer treatment. Finally, we investigated whether the patients had received any adjuvant therapy after the bronchoscopic intervention.

### Statistical analysis

Data are presented as number (%) for categorical variables and median (interquartile range [IQR]) for continuous variables. The Kaplan-Meier method was used to estimate the overall survival rate after the first bronchoscopic intervention. A multivariable Cox proportional hazard regression analysis with backward stepwise selection, with *P* <  0.05 for entry of variables, and *P* > 0.10 for removal of variables were used to identify independent prognostic factors associated with overall survival. Statistical differences were considered significant at *P* <  0.05. All statistical analyses were performed using SPSS software (IBM SPSS Statistics ver. 25, Chicago, IL, USA).

## Results

### Baseline characteristics

The characteristics of the study population are described in Table 1. Among 224 patients, 191 (85.3%) were male, and the median age was 63 (IQR, 56–69) years. Of these patients, 176 (78.6%) had a smoking history of median 40 (IQR, 30–50) pack-year. The most common comorbidity was chronic pulmonary disease (20.1%). Sixty-eight patients (30.4%) had a poor performance status, and intubation was required before the intervention in 27 (12.1%) patients. Squamous cell carcinoma (71.0%) was the most common histological type of malignancy.

**Table 1:** Baseline characteristics

Variables	*N* = 224
Age, years	63 (56–69)
Sex, male	191 (85.3)
Body mass index, kg/m^2^	21.9 (20.1–24.1)
Smoking	
Never smoker	48 (21.4)
Ex-smoker	102 (45.5)
Current smoker	74 (33.0)
Pack-year (*n* = 166)^a^	40 (30–50)
Comorbidity	
Chronic pulmonary disease	45 (20.1)
Diabetes mellitus	31 (13.8)
Congestive heart disease	15 (6.7)
Chronic liver disease	10 (4.5)
Chronic kidney disease	5 (2.2)
Cerebrovascular disease	2 (0.9)
Poor performance status^b^	68 (30.4)
Intubation due to respiratory failure before intervention	27 (12.1)
Histological type of malignancy	
Non-small cell carcinoma	215 (96.0)
Squamous cell carcinoma	159 (71.0)
Adenocarcinoma	40 (17.9)
Others^c^	16 (7.1)
Small cell carcinoma	9 (4.0)

Data are presented as *n* (%) or the median (interquartile range)

^a^ Excluding never smoker and 11 patients with ex-smoker who had no information

^b^ American Society of Anesthesiologists (ASA) physical status class ≥3 means severe systemic disease with functional limitation

^c^ Poorly differentiated (*n* = 7), large cell neuroendocrine carcinoma (*n* = 3), pleomorphic carcinoma (*n* = 3), lymphoepithelioma-like carcinoma (*n* = 1), sarcomatoid carcinoma (*n* = 1), basaloid squamous cell carcinoma (*n* = 1)

Characteristics of the MCAO site are described in Table 2. Among 224 patients, 173 (77.2%) had a single-site obstruction, while 51 (22.8%) had obstructions at two or more sites. Endobronchial, mixed, and extrinsic types of obstructions were identified in 125 (55.8%), 80 (35.7%), and 19 (8.5%) patients, respectively. Most patients (76.8%) had ≥71% obstruction of the cross-sectional area (Myer and Cotton Grade III or IV). The median length of stenosis was 27 mm (IQR, 18–35 mm). Some patients (2.2%) had a fistula between the trachea and esophagus.

**Table 2:** Bronchoscopic findings

Variables	*N* = 224
Site of lesion	
Single lesion	173 (77.2)
Trachea	44 (19.6)
Left main bronchus	45 (20.1)
Right main bronchus	37 (16.5)
Right bronchus intermedius	20 (8.9)
Lobar bronchus	27 (12.1)
Extended lesion	51 (22.8)
Trachea and each or both bronchi	26 (11.6)
Both bronchi	25 (11.2)
Type of obstruction	
Endobronchial lesion	125 (55.8)
Extrinsic compression	19 (8.5)
Mixed lesion	80 (35.7)
Severity of stenosis (Myer and Cotton grade)^a^	
II	52 (23.1)
III	97 (43.4)
IV	75 (33.5)
Length of MCAO^b^, mm	27 (18–35)
Combined fistula from trachea to esophagus	5 (2.2)

Data are presented as *n* (%) or the median (interquartile range)

*MCAO* malignant central airway obstruction

^a^Categorization based on the percentage of reduction in cross-sectional area. Grade 1, ≤ 50% lumenal stenosis; Grade II, 51–70% lumenal stenosis; Grade III, 71–99% lumenal stenosis; Grade IV, no lumen

^b^Length of MCAO was defined as the sum of the length of the obstructive lesions more than grade II

### Treatment modalities and complications

Treatment-related information is detailed in Table 3. There was an average of 0.5 months (IQR, 0.2–1.3 months) from diagnosis of MCAO to the first bronchoscopic intervention. The median procedure time was 35 min (IQR, 28–45 min). Mechanical debulking was performed in patients who had endobronchial and mixed lesions; additional procedures, such as stent insertion (50.4%), laser cauterization (31.7%), and ballooning (10.3%), were used in combination according to the characteristics of the individual lesions. During the study period, 55 (24.6%) patients received two or more bronchoscopic interventions. Overall, technical success was achieved in 93.7%. Of the 96 (42.9%) patients who were diagnosed with MCAO during initial diagnosis of malignancy, 27 (12.1%) did not receive adjuvant treatment after interventional bronchoscopy because of poor general condition (*n* = 23) or rejection of additional treatment by the patient (*n* = 4). Of 128 (57.1%) patients diagnosed with MCAO as a part of the disease progression of malignancy, 42 (18.7%) did not receive adjuvant treatment after interventional bronchoscopy due to of poor general condition (*n* = 32), rejection of additional treatment (*n* = 7), or lack of further treatment options (*n* = 3).

**Table 3:** Treatment modalities and complications

Variables	*N* = 224
Time interval from diagnosis of MCAO to treatment, months	0.5 (0.2–1.3)
Procedure time, min	35 (28–45)
Treatment modalities	
Mechanical debulking	205 (91.5)
Silicone stent	113 (50.4)
Tube stent	96 (42.9)
Y stent	17 (7.6)
Laser	71 (31.7)
Ballooning	23 (10.3)
Number of interventional bronchoscopies	
1	169 (75.4)
≥ 2	55 (24.6)
Technical failure	14 (6.3)
MCAO as initial diagnosis of malignancy	96 (42.9)
No adjuvant treatment after interventional bronchoscopy	27 (12.1)
Adjuvant radiation therapy	44 (19.6)
Adjuvant chemotherapy	31 (13.8)
Adjuvant surgical resection	19 (8.5)
MCAO as disease progression of malignancy	128 (57.1)
No adjuvant treatment after interventional bronchoscopy	42 (18.8)
Adjuvant radiation therapy	49 (21.9)
Adjuvant chemotherapy	56 (25.0)
Adjuvant surgical resection	6 (2.7)
Acute complications	33 (17.0)
Respiratory distress	18 (8.0)
Excessive bleeding	14 (6.3)
Pneumothorax	3 (1.3)
Procedure-related death^a^	3 (1.3)
Chronic complications	48 (21.4)
Granulation tissue overgrowth	26 (11.6)
Restenosis	20 (8.9)
Mucostasis	16 (7.1)
Stent migration	3 (1.3)

Data are presented as *n* (%) or the median (interquartile range)

*MCAO* malignant central airway obstruction

Patients could undergo more than one adjuvant treatment

Patients could have one more complications

^a^ Three patients died of excessive bleeding (*n* = 2) and tension pneumothorax (*n* = 1)

Acute complications occurred in 33 (17%) patients, comprising excessive bleeding (*n* = 14), respiratory distress (*n* = 18), and pneumothorax (*n* = 3). Three procedure-related deaths were recorded due to excessive bleeding (*n* = 2) and tension pneumothorax (*n* = 1). Although chronic complications occurred in 48 (21.4%) patients, including granulation tissue overgrowth (*n* = 26), restenosis (*n* = 20), mucostasis (*n* = 16), and stent migration (*n* = 3), most complications were manageable with additional procedures.

### Survival and prognosis

Table 4 shows the independent prognostic factors related to mortality based on univariate and multivariate Cox proportional hazard regressions. First, of the host-related factors, chronic pulmonary disease (adjusted hazard ratio [aHR], 1.640; 95% confidence interval [CI], 1.082–2.488; *P* = 0.020) and poor performance status (aHR, 1.750; 95% CI, 1.206–2.540; *P* = 0.003) were associated with poor survival. Second, of the lesion-related factors, an extended lesion was significantly associated with worse survival compared to a single lesion (aHR, 1.545; 95% CI, 1.035–2.305; *P* = 0.033). In addition, extrinsic compression (aHR, 2.119; 95% CI, 1.120–4.011; *P* = 0.021) and mixed lesions (aHR, 2.388; 95% CI, 1.657–3.442; *P* <  0.001) had a lower survival rate than endobronchial lesions. Finally, of the disease status and adjuvant treatment-related factors, patients with MCAO as disease progression without adjuvant treatment (aHR, 5.099; 95% CI, 3.075–8.453; *P* <  0.001) had the worst survival among all the patient groups. Patients who were diagnosed with MCAO during initial diagnosis but did not receive adjuvant treatment (aHR, 2.370; 95% CI, 1.349–4.162; *P* = 0.003) and patients identified as having MCAO during disease progression who did receive adjuvant treatments (aHR, 2.179; 95% CI, 1.413–3.359; *P* <  0.001) had similar worse prognoses than those diagnosed with MCAO during initial diagnosis who received adjuvant treatments.

**Table 4:** Prognostic factors related to mortality (*n* = 224)

Variable	Univariable Cox regression		Multivariable Cox regression	
	Unadjusted HR (95% CI)	*P*	Adjusted HR (95% CI)	*P*
***Host-related factors***				
Age, years	1.018 (0.997–1.039)	0.090		
Sex, male	1.192 (0.721–1.969)	0.493		
Body mass index, kg/m^2^	0.936 (0.887–0.987)	0.015		
Smoking history				
No	Reference			
Yes	1.062 (0.709–1.589)	0.772		
Comorbidity				
Chronic pulmonary disease	1.764 (1.135–2.740)	0.012	1.640 (1.082–2.488)	0.020
Diabetes mellitus	1.625 (0.989–2.670)	0.055		
Congestive heart disease	0.719 (0.369–1.401)	0.332		
Chronic liver disease	0.458 (0.194–1.082)	0.075	0.529 (0.231–1.211)	0.132
Chronic kidney disease	1.723 (0.488–6.081)	0.398		
Cerebrovascular disease	0.616 (0.077–4.913)	0.647		
Poor performance status^a^	1.946 (1.276–2.968)	0.002	1.750 (1.206–2.540)	0.003
Intubation due to respiratory failure before intervention	0.881 (0.500–1.553)	0.661		
Histological type of malignancy				
Adenocarcinoma	Reference			
Squamous cell carcinoma	1.075 (0.662–1.746)	0.769		
Others^b^	1.306 (0.614–2.777)	0.489		
Small cell carcinoma	1.863 (0.750–4.627)	0.180		
***Lesion-related factors***				
Site of lesion				
Single lesion	Reference		Reference	
Extended lesion	1.399 (0.903–2.167)	0.133	1.545 (1.035–2.305)	0.033
Type of obstruction				
Endobronchial lesion	Reference		Reference	
Extrinsic compression	2.525 (1.245–5.125)	0.010	2.119 (1.120–4.011)	0.021
Mixed lesion	2.555 (1.685–3.874)	< 0.001	2.388 (1.657–3.442)	< 0.001
Severity of stenosis (Myer and Cotton grade)^c^				
II and III	Reference			
IV	1.272 (0.887–1.824)	0.191		
Length of MCAO^d^, mm	1.002 (0.988–1.016)	0.787		
Combined fistula	0.487 (0.157–1.511)	0.213		
***Disease status and adjuvant treatment-related factors***				
MCAO as initial diagnosis with adjuvant treatment	Reference		Reference	
MCAO as initial diagnosis without adjuvant treatment	2.344 (1.318–4.169)	0.004	2.370 (1.349–4.162)	0.003
MCAO as disease progression with adjuvant treatment	2.122 (1.352–3.332)	0.001	2.179 (1.413–3.359)	< 0.001
MCAO as disease progression without adjuvant treatment	5.296 (3.142–8.926)	< 0.001	5.099 (3.075–8.453)	< 0.001

*HR* hazard ratio; *CI* confidential interval; *MCAO* malignant central airway obstruction

^a^ American Society of Anesthesiologists (ASA) physical status grade ≥ 3 means severe systemic disease with functional limitation

^b^ Poorly differentiated (*n* = 7), large cell neuroendocrine carcinoma (*n* = 3), pleomorphic carcinoma (*n* = 3), lymphoepithelioma-like carcinoma (*n* = 1), sarcomatoid carcinoma (*n* = 1), basaloid squamous cell carcinoma (*n* = 1)

^c^ Categorization based on the percentage of reduction in cross-sectional area. Grade 1, ≤ 50% lumenal stenosis; Grade II, 51–70% lumenal stenosis; Grade III, 71–99% luminal stenosis; Grade IV, no lumen

^d^ Length of MCAO was defined as the sum of the length of the obstructive lesions more than grade II

**Fig. 1:**
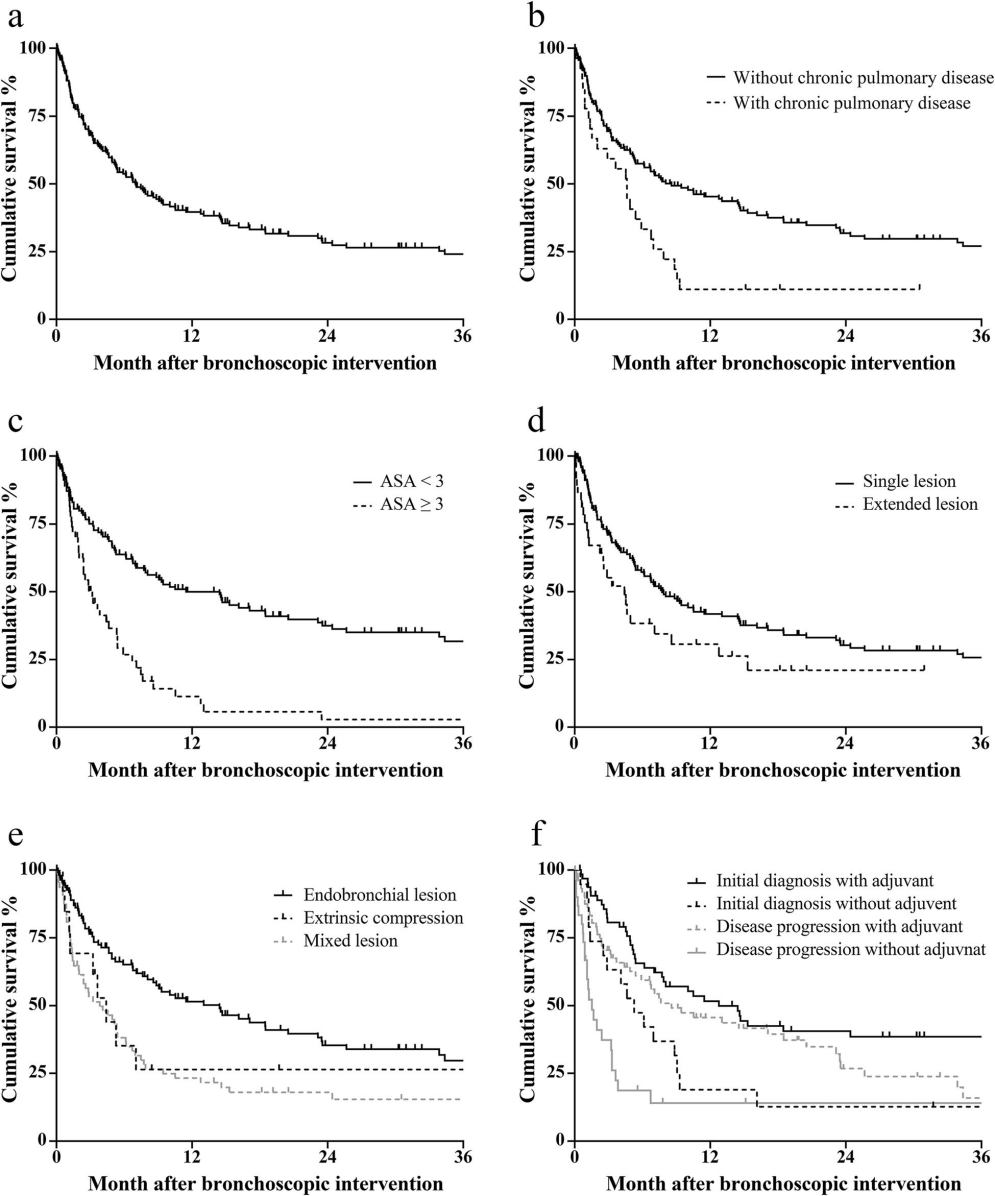
The overall survival rate of patients after interventional bronchoscopy. Survival rates based on (**a**) total participants, (**b**) chronic pulmonary disease, (**c**) poor performance status, (**d**) site of the lesion, (**e**) type of obstruction, and (**f**) detection time of MCAO and possibility of adjuvant treatment after interventional bronchoscopy. MCAO, malignant central airway obstruction

The overall survival period had a median of 7.0 months; the one-year and two-year survival rates were 39.7 and 28.3%, respectively (Fig. 1a). Figure 1 also shows the overall survival after the first bronchoscopic intervention according to independent prognostic factors (existence of chronic pulmonary disease, performance status, site of lesion, type of obstruction, and detection time for MCAO with or without adjuvant treatment).

## Discussion

In this study, technical success of interventional bronchoscopy was achieved in 93.7% of patients with MCAO due to primary pulmonary malignancy, and the procedure-related death rate was only 1.3%. An important point of our study is that we tried to identify the independent prognostic factors associated with survival after the first bronchoscopic intervention.

Most patients with primary pulmonary malignancy die from disease progression or complications during treatment, and MCAO is one of the major complications causing death [4, 5]. Interventional bronchoscopy is gradually being considered a safe procedure and can provide immediate symptomatic relief of dyspnea [14, 24]. In the long-term, primary pulmonary malignancy patients with MCAO treated by bronchoscopic intervention have similar survival rates to those without MCAO [25]. Recent studies have confirmed the safety and efficacy of interventional bronchoscopy, and its role as a treatment modality is expected to increase over time [14, 26]. However, considering the risk of general anesthesia during the procedure, complications, and failure rate around 5%, it is essential to identify the best candidates for interventional bronchoscopy [27, 28].

We investigated the prognostic factors that affect survival after bronchoscopic intervention. First, of the host-related factors, poor performance status and chronic pulmonary disease were associated with poor survival. Patient general health status was reported by previous studies to be a representative factor [13, 27]. As patient performance status decreases, procedure-related complications, such as pneumonia and respiratory distress, may increase, and the opportunity for adjuvant treatment after interventional bronchoscopy may decrease [14]. In addition, smoking status and chronic pulmonary disease were risk factors of complications after bronchoscopic intervention [29]. Therefore, we think poor performance status and chronic pulmonary disease might be independently associated with survival after the first bronchoscopic intervention.

Second, lesion-related factors are degree of extension and type of obstruction at the lesion. In a previous study, patients with single lesion restricted to one lung demonstrated a better survival than those with tracheal obstruction and involvement of both bronchi [30]. Our study showed that extensive lesion was one of the prognostic factors for poor survival. In the case of single lesions, airway patency could be maintained by stenting or other treatment modalities until adjuvant treatments became available. However, in the case of extension lesions, it is possible that the MCAO may recur before adjuvant treatment can begin. The frequency of complications may also increase with repeated obstructions. Endobronchial lesions can be treated by mechanical debulking or laser cauterization, but mixed lesions require multimodality therapy to maintain airway patency [31]. Multimodality therapy often increases the incidence of procedure-related complications and patient mortality [27, 28]. In patients with extrinsic compression, stent insertion is needed to maintain airway patency, but stent placement was one of the risk factors of post-intervention complications and was correlated with poorer survival [27, 32]. In this study, a mixed lesion and extrinsic compression were also prognostic factors of poor survival and could be explained using the reasons described above.

Finally, disease status and adjuvant treatment-related factors are detection time of MCAO and possibility of adjuvant treatment. Many studies have addressed the correlation between additional therapy and survival. Adjuvant treatment after bronchoscopic intervention is known to be associated with a better survival rate [7, 12, 21]. In addition, several studies have explored the impact of previous history of anti-cancer therapy. Jeon et al. demonstrated that newly diagnosed and untreated MCAO patients had a longer survival time following bronchoscopic intervention, and Perin et al. reported that previous chemotherapy was a potential risk factor of intervention-related complications [12, 29]. In contrast, Guibert et al. showed that patients who had been previously treated for MCAO had a better survival rate [13]. Despite these results, Guibert et al. recommended that bronchoscopic intervention should be considered as an early multimodal treatment, not a last action. Because they identified a selection bias among previously untreated patients who received fewer additional treatments after intervention. In our study, patients who were initially diagnosed with MCAO and had undergone adjuvant therapy had the best survival rate among all the patient groups. MCAO caused by disease progression with adjuvant therapy had a similar prognosis as initially diagnosed MCAO without adjuvant therapy. These results suggest that possibility of adjuvant therapy and initial diagnosis are important factors related to survival.

Certain limitations of our study must be acknowledged. First, this was a retrospective study with a single-institution design, which is a source of selection bias. Especially, the proportion of chronic pulmonary disease was reported to 20% in this retrospective study, which is far lower than 30–35% of the largest multicenter cohort study for patients with malignant central airway obstruction [14]. Therefore, the effect of comorbidities may not be properly assessed on the prognosis. Second, treatment options for patients in the terminal stage of primary pulmonary malignancy, such as chemoradiotherapy and supportive care, have advanced over time. These developments might have influenced the slower disease progression and better survival rates of patients in the later study phase compared with those in the earlier study phase. Finally, we were unable to obtain patient spirometric data, quality of life, and symptom scores before and after the bronchoscopic intervention. Most procedures for MCAO patients were provided as palliative therapy, so symptom scores or quality of life assessments might be important factors to consider in this patient population.

## Conclusions

In conclusion, bronchoscopic intervention could be a safe and effective procedure for patients with MCAO due to primary pulmonary malignancy. In addition, a poor survival rate was associated with chronic pulmonary disease, poor performance status, extended lesion (vs. single lesion), mixed lesion or extrinsic compression (vs. endobronchial lesion), and MCAO detected as part of the disease progression and/or not receiving adjuvant treatment after the bronchoscopic intervention. This study will help clinicians to determine the best candidates for bronchoscopic intervention among patients with MCAO due to primary pulmonary malignancy.

## Data Availability

The datasets used and/or analyzed during the current study are available from the corresponding author on reasonable request.

## Abbreviations

aHR: Adjusted hazard ratio
ASA: American Society of Anesthesiologists
CI: Confidence interval
CT: Computed tomography
IQR: Interquartile range
MCAO: Malignant central airway obstruction

## References

[1] Johnson DH, Schiller JH, Bunn PA (2014). Recent clinical advances in lung cancer management. J Clin Oncol.

[2] Reck M, Heigener DF, Mok T, Soria JC, Rabe KF (2013). Management of non-small-cell lung cancer: recent developments. Lancet..

[3] Forde PM, Ettinger DS (2013). Targeted therapy for non-small-cell lung cancer: past, present and future. Expert Rev Anticancer Ther.

[4] Bray F, Ferlay J, Soerjomataram I, Siegel RL, Torre LA, Jemal A (2018). Global cancer statistics 2018: GLOBOCAN estimates of incidence and mortality worldwide for 36 cancers in 185 countries. CA Cancer J Clin.

[5] Torre Lindsey A., Siegel Rebecca L., Jemal Ahmedin (2015). Lung Cancer Statistics. Lung Cancer and Personalized Medicine.

[6] Ernst A, Feller-Kopman D, Becker HD, Mehta AC (2004). Central airway obstruction. Am J Respir Crit Care Med.

[7] Saji H, Furukawa K, Tsutsui H, Tsuboi M, Ichinose S, Usuda J (2010). Outcomes of airway stenting for advanced lung cancer with central airway obstruction. Interact Cardiovasc Thorac Surg.

[8] Wood DE (2002). Management of malignant tracheobronchial obstruction. Surg Clin North Am.

[9] Nihei K, Ishikura S, Kawashima M, Ogino T, Ito Y, Ikeda H (2002). Short-course palliative radiotherapy for airway stenosis in non-small cell lung cancer. Int J Clin Oncol.

[10] Santos RS, Raftopoulos Y, Keenan RJ, Halal A, Maley RH, Landreneau RJ (2004). Bronchoscopic palliation of primary lung cancer: single or multimodality therapy?. Surg Endosc.

[11] Seijo LM, Sterman DH (2001). Interventional pulmonology. N Engl J Med.

[12] Jeon K, Kim H, Yu CM, Koh WJ, Suh GY, Chung MP (2006). Rigid bronchoscopic intervention in patients with respiratory failure caused by malignant central airway obstruction. J Thorac Oncol.

[13] Guibert N, Mazieres J, Lepage B, Plat G, Didier A, Hermant C (2014). Prognostic factors associated with interventional bronchoscopy in lung cancer. Ann Thorac Surg.

[14] Ost DE, Ernst A, Grosu HB, Lei X, Diaz-Mendoza J, Slade M (2015). Therapeutic bronchoscopy for malignant central airway obstruction: success rates and impact on dyspnea and quality of life. Chest..

[15] Lee JH, Jung EJ, Jeon K, Koh WJ, Suh GY, Chung MP (2011). Treatment outcomes of patients with adenoid cystic carcinoma of the airway. Lung Cancer.

[16] Molina JR, Aubry MC, Lewis JE, Wampfler JA, Williams BA, Midthun DE (2007). Primary salivary gland-type lung cancer: spectrum of clinical presentation, histopathologic and prognostic factors. Cancer..

[17] Chen H, Zhang J, Qiu XJ, Wang J, Pei YH, Wang YL (2017). Interventional Bronchoscopic therapy in adult patients with tracheobronchial Mucoepidermoid carcinoma. Chin Med J.

[18] Fulford LG, Kamata Y, Okudera K, Dawson A, Corrin B, Sheppard MN (2001). Epithelial-myoepithelial carcinomas of the bronchus. Am J Surg Pathol.

[19] Kim H (1998). Stenting therapy for stenosing airway disease. Respirology..

[20] Colt HG, Dumon JF (1995). Airway stents. Present and future Clin Chest Med.

[21] Shin B, Chang B, Kim H, Jeong BH (2018). Interventional bronchoscopy in malignant central airway obstruction by extra-pulmonary malignancy. BMC Pulm Med.

[22] Owens WD, Felts JA, Spitznagel EL (1978). ASA physical status classifications: a study of consistency of ratings. Anesthesiol..

[23] Myer CM, O'Connor DM, Cotton RT (1994). Proposed grading system for subglottic stenosis based on endotracheal tube sizes. Ann Otol Rhinol Laryngol.

[24] Oviatt PL, Stather DR, Michaud G, Maceachern P, Tremblay A (2011). Exercise capacity, lung function, and quality of life after interventional bronchoscopy. J Thorac Oncol.

[25] Chhajed PN, Baty F, Pless M, Somandin S, Tamm M, Brutsche MH (2006). Outcome of treated advanced non-small cell lung cancer with and without central airway obstruction. Chest..

[26] Guibert N, Mhanna L, Droneau S, Plat G, Didier A, Mazieres J (2016). Techniques of endoscopic airway tumor treatment. J Thorac Dis.

[27] Ost DE, Ernst A, Grosu HB, Lei X, Diaz-Mendoza J, Slade M (2015). Complications following therapeutic bronchoscopy for malignant central airway obstruction: results of the AQuIRE registry. Chest..

[28] Grosu HB, Eapen GA, Morice RC, Jimenez CA, Casal RF, Almeida FA (2013). Stents are associated with increased risk of respiratory infections in patients undergoing airway interventions for malignant airways disease. Chest..

[29] Perin B, Zaric B, Jovanovic S, Matijasevic J, Stanic J, Kopitovic I (2012). Patient-related independent clinical risk factors for early complications following Nd: YAG laser resection of lung cancer. Ann Thorac Med.

[30] Chhajed PN, Somandin S, Baty F, Mehta AJ, Azzola A, Leuppi J (2010). Therapeutic bronchoscopy for malignant airway stenoses: choice of modality and survival. J Cancer Res Ther.

[31] Mudambi L, Miller R, Eapen GA (2017). Malignant central airway obstruction. J Thorac Dis..

[32] Verma A, Goh SK, Tai DYH, Kor AC, Abisheganaden J, Sein ZNN (2019). Outcome differences between recanalized malignant central airway obstruction from endoluminal disease versus extrinsic compression. Lasers Med Sci.

